# Effect of individually tailored nutritional counseling on frailty status in older adults with protein-energy malnutrition or risk of it: an intervention study among home care clients

**DOI:** 10.1038/s41430-024-01547-0

**Published:** 2024-11-23

**Authors:** Tarja Kaipainen, Sirpa Hartikainen, Miia Tiihonen, Irma Nykänen

**Affiliations:** 1 Kuopio Research Centre of Geriatric Care, School of Pharmacy, Faculty of Health Sciences, University of Eastern Finland, Kuopio, Finland; 2 Institute of Public Health and Clinical Nutrition, School of Medicine, Faculty of Health Sciences, University of Eastern Finland, Kuopio, Finland

**Keywords:** Nutrition, Geriatrics

## Abstract

**Background:**

Frailty and protein-energy malnutrition (PEM) are common in older home care clients. In this study, we evaluate the effect of individually tailored dietary counseling on frailty status among home care clients with PEM or its risk aged 75 or older with a follow-up of six months.

**Methods:**

This intervention study is part of the non-randomized population-based Nutrition, Oral Health and Medication (NutOrMed) study in Finland. The frailty was assessed using the abbreviated Comprehensive Geriatric Assessment (aCGA) and included 15 questions from three different domains: cognitive status (MMSE), functional status (ADL, IADL) and depression (GDS-15). The study population consisted of persons with PEM or its risk (intervention group *n* = 90, control group *n* = 55). PEM or its risk was defined by MNA score <24 and/or plasma albumin <35 g/l. Registered nutritionist gave individually tailored nutritional counseling for participants at the baseline and nutritional treatment included conventional food items.

**Results:**

The mean age was 83.9 in the intervention and 84.3 in the control group. At the baseline frailty prevalence was 74.4% (*n* = 67) and after six-month 61.1% (*n* = 55) in the intervention group and, respectively 74.5% (*n* = 41) and 80.0% (*n* = 44) in the control group. The intervention decreased significantly (*p* < 0.001) the prevalence of frailty in the intervention group, while it increased in the control group.

**Conclusions:**

Individually tailored nutritional counseling reduces the prevalence of frailty among vulnerable home care clients with PEM or its risk. In the nutritional treatment of frailty, adequate intake of protein and energy should be a cornerstone of treatment.

## Introduction

Frailty is a syndrome commonly found in older people [1], characterized by reduced physiological resources and resilience to stressors [2]. This geriatric condition makes older people more susceptible to adverse health effects and increases the risk of functional inability, immobility, falls, loneliness, social isolation, disability, hospitalization, institutionalization, mortality [1–4], and healthcare costs [5]. The prevalence of frailty among older home care clients ranges from 41.5 to 90% [6–8].

Protein-energy malnutrition (PEM) is also a significant problem among older people [9, 10]. PEM is a form of malnutrition when the protein and energy intake do not meet the nutritional requirements [11]. Frailty and PEM are closely linked, with each having an impact on the other [12, 13]. Frail individuals are more likely to have PEM, and PEM increases the risk of frailty and the negative outcomes associated with it [6, 14, 15]. However, frailty and PEM are potentially reversible situations, so there is a need for clinical interventions focusing on treating PEM and investigating whether it affects frailty status.

In our previous study, we found that it is possible to improve the nutritional status of older home care clients by providing individually tailored nutritional counseling that takes into account their eating preferences [16]. However, we have little knowledge about the effect of nutritional intervention on frailty status. Therefore, we aimed to study the effect of individually tailored dietary counseling on frailty status among home care clients aged 75 years or over with PEM or at risk of it with a follow-up of six months.

## Methods

### Design and participants

This intervention study is a part of the population-based multidisciplinary Nutrition, Oral Health and Medication study (NutOrMed study), which aimed to evaluate and improve the nutritional status, oral health, and functional ability of older home care clients in three cities in Eastern and Central Finland [17]. A detailed description of the NutOrMed study and its design is described in the previous article [17]. In Finland, municipalities and private organizations offer home care services, including home help, nursing, management of medications, and medical care.

This study included a subpopulation of home care clients with PEM or its risk. The intervention group included 90 participants, and the control group 55 participants. To prevent any potential influence, the intervention group was located approximately 100 km away from the towns of the control groups. There were no exclusion criteria based on morbidity or cognitive status. All the participants were able to eat normal food items. If a participant was unable to reply, typically due to cognitive impairment, the data collection was supplemented by a caregiver or home care nurse.

### Measurements

Frailty was assessed using the abbreviated Comprehensive Geriatric Assessment (aCGA), which includes three questions on Activities of Daily Living (ADL), four questions on Instrumental Activities of Daily Living (IADL), four questions on the Mini Mental State Examination (MMSE), and four questions on the Geriatric Depression Scale (GDS-15) [18]. According aCGA frailty was defined when the cut-off value was exceeded in at least two domains (function ability, cognitive symptoms, depressive symptoms) on the aCGA (Supplementary Table 1). The cut-off value for function ability (ADL, IADL) was ≥1, for cognitive symptoms (MMSE), ≤6, and for depressive symptoms (GDS-15) ≥ 2. The validated aCGA is designed to identify frailty in vulnerable individuals and it can detect problems in daily living such as difficulties in bathing, dressing and shopping as well as declined cognition or depressive symptoms [18]. The participants’ nutritional status was assessed using a Mini Nutritional Assessment (MNA) by a clinical nutritionist. The MNA is a validated and standardized tool for detecting the nutritional status of older people [19–22]. Both PEM and the risk of PEM were defined as a score of less than 24 on the MNA and/or plasma albumin less than 35 g/l [18].

Drug use was defined as regular and as-needed use of prescription and over-the-counter drugs. Polypharmacy was defined as the use of 10 or more drugs regularly or as needed [23]. Oral health was evaluated by a dental hygienist. The feeling of dry mouth was assessed by asking the participants if they had experienced it, and the responses were categorized as none, occasionally or continuously. Chewing problems were evaluated with the question “Do you have chewing problems?” with the answer options being “yes” or “no”.

The information on cardiovascular diseases, diabetes, asthma/COPD, stroke, visual or hearing impairment, and depressive symptoms was obtained from electronic medical records based on a clinical diagnosis. More comorbidities were defined by the Functional Comorbidity Index (FCI) (including 13 diseases) based on health records [24]. The diagnosis of any disorder, including cognitive disorder, was verified by a geriatrician through medical records. Functional ability was assessed using the ADL [25] scale, with a cutoff score of <80, and the IADL [26] scale, with a cutoff score of ≤4. Cognition was assessed using the Mini-Mental State Examination (MMSE) [27], with a cutoff score of ≤24. The ability to walk 400 meters was self-reported by the participants, with categories ranging from unable to walk to able to walk independently with or without difficulties. Participants were asked to rate their current health status into two categories: quite good and good, or average, quite bad, and bad. Demographic data was also collected at baseline. All measurements were taken at baseline and after a six-month follow-up, except for drug use and comorbidities, which were only evaluated at baseline. In cases where participants were unable to respond, data was supplemented by a caregiver or nurse. More details of the measurements have been described in a previous study [17].

### Intervention

The nutritional intervention was based on the results of the baseline MNA test, plasma albumin, and a 24-h dietary recall. For the intervention group, a clinical nutritionist provided one individualized nutritional guidance based on the 24-h dietary recall and planned individualized nutritional care during the same visit, in collaboration with the client and their nurse or family members. The nutritional care plan was based on international recommendations for protein intake [28] and national nutrition recommendations for older persons [29]. The goal for protein intake was 1 g/kg, and energy intake was 125,6 kJ/kg per day. The intervention aimed to increase the intake of energy-dense foods, meals, and protein- and nutrient-rich snacks with normal food items. While a vitamin D supplement was recommended, it was not included in the analysis, and no multivitamin supplements were prescribed. In case the participant had cognitive decline or other reasons why he/she had difficulties understanding, remembering, or following up, nutritional counseling was carried out in close cooperation with the home care nurse or caregiver. The control group did not receive any nutritional guidance. More details of the nutritional intervention have been described in a previous study [30]. The effects of the intervention were monitored after six months follow-up in both study groups.

### Statistical analysis

Group characteristics were compared using independent samples *t* test (normally distributed outcomes) or Pearson’s Chi-square test as appropriate. The normality of variables was checked with Shapiro–Wilk’s test. To compare the effect of the intervention between the groups, a generalized estimation equations model analysis was adopted, which was adjusted for age, gender, education years, and cognitive decline. Improvements in frailty status and protein and energy intake during the intervention were analyzed using a mixed-effect regression model. Participants assessed at baseline and after six months were included in the data analysis (per protocol). Any *p* value less than 0.05 was statistically significant. The data was analyzed using SPSS 27.0 software (IBM Corp., Armonk, NY, USA).

## Results

The study involved participants with a mean age of 83.9 years, with 72.2% of them being female in the intervention group, and 84.3 years and 63.6% in the control group respectively (Table 1). The intervention group had more years of education and a greater intake of energy (kJ/kg/d) and protein (g/kg/day), as well as more frequent excessive polypharmacy and strokes, and lower FCI and cognition compared to the control group. After the intervention, the intervention group had a higher MNA score and less PEM or its risk, as well as a greater intake of energy (kJ/kg/d) and protein (g/kg/day), and more participants were able to walk 400 meters compared to the control group.

**Table 1:** Characteristics of participants with protein-energy malnutrition (PEM) at baseline and the change in nutritional status and functional ability after 6 months of follow-up in the intervention and control group.

	Intervention group *n* = 90	Control group *n* = 55	*P*-value	Intervention group *n* = 90 6 mo	Control group *n* = 55 6 mo	*P*-value
Demographic						
Female, *n* (%)	65 (72.2)	35 (63.6)	0.278			
Age, mean (SD)	83.9 (5.1)	84.3 (5.4)	0.669			
Living alone, *n* (%)	55 (61.1)	36 (65.5)	0.658			
Education, year (SD)	9.3 (4.1)	6.9 (1.8)	<0.001			
Nutritional status						
MNA, mean (SD)	21.5 (2.1)	21.6 (2.5)	0.804	25.8 (2.6)	23.8 (3.0)	<0.001
≤23.5, *n* (%)	88 (97.8)	53 (96.4)	0.614	15 (16.7)	20 (36.3)	0.011
Protein g/kg/day	0.90	0.74	<0.001	1.0	0.8	<0.001
Energy kJ/kg/day	95.04	74.9	<0.001	95.73	82.5	0.007
Functional ability						
ADL < 80, *n* (%)	23 (25.6)	8 (14.5)	0.117	23 (25.6)	11 (20)	0.444
IADL ≤ 4, *n* (%)	32 (35.6)	24 (43.6)	0.250	45 (50)	36 (65.6)	0.069
Able to walk 400 m, *n* (%)	56 (62.2)	35 (63.6)	0.931	59 (65.6)	27 (49.1)	0.041
Poor self-rated health, n (%)	23 (25.6)	20 (36.4)	0.180	24 (26.7)	19 (34.5)	0.325
MMSE ≤ 24, *n* (%)	26 (28.9)	30 (54.5)	0.002			
Excessive polypharmacy (%)	48 (53.3)	39 (70.9)	0.036			
Oral Health						
Dry mouth, *n* (%)	52 (57.8)	30 (54.5)	0.648			
Chewing problems, *n* (%)	17 (18.9)	8 (14.5)	0.358			
Comorbidities						
Cardiovascular disease, *n* (%)	53 (58.9)	32 (58.2)	0.680			
Diabetes, *n* (%)	28 (31.1)	12 (21.8)	0.316			
Asthma/COPD, *n* (%)	21 (23.3)	8 (14.5)	0.266			
Stroke, *n* (%)	25 (27.8)	7 (12.7)	0.050			
Visual or hearing impairment, *n* (%)	29 (32.2)	15 (27.3)	0.701			
Depressive symptoms, *n* (%)	27 (30.0)	19 (34.5)	0.568			
FCI, mean (SD)	2.2 (1.6)	4.1 (1.9)	<0.001			

*mo* month, *SD* Standard deviation, *MNA* Mini Nutritional Assessment, *ADL* Activities of Daily Living (Barthel Index), *IADL* Instrumental Activities of Daily Living, *MMSE* Mini Mental State Examination, *Excessive polypharmacy* 10≥ drugs in use, *COPD* Chronic obstructive pulmonary disease, *FCI* Functional Comorbidity Index.

**Fig. 1:**
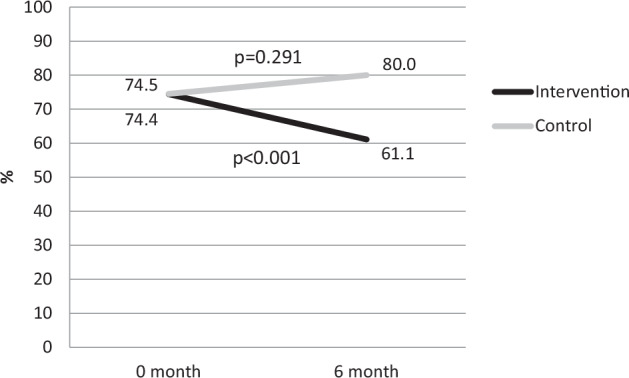
Proportions (%) of participants with frailty in the intervention and control group at baseline and after 6 months of follow-up. Panel 0 month represents the baseline data, and panel 6 month represents the follow-up data. *p* < 0.05 indicates a statistically significant difference within the same group over time.

At baseline, the prevalence of frailty in the intervention and control groups did not differ (*p* = 0.847). At baseline, in the intervention group, the prevalence of frailty was 74.4% (*n* = 67), and after six months, it decreased significantly to 61.1% (*n* = 55) (*p* < 0.001) (Table 2, Fig. 1). In the control group, the prevalence of frailty increased from 74.5% (*n* = 41) to 80.0% (*n* = 44) (*p* = 0.291). After the intervention, there was a significant difference in the prevalence of frailty between the study groups (*p* = 0.047). Furthermore, the higher protein (g/kg/d) and energy (kJ/kg/d) intake of the intervention group were significantly associated with the decrease in frailty status when adjusted by age, sex, education, excessive polypharmacy, and FCI (*p* = 0.009 and *p* = 0.023, respectively). Additionally, the prevalence of depressive symptoms domain of aCGA decreased during the study in both groups (Table 2).

**Table 2:** Prevalence of function ability, cognitive and depressive symptoms in abbreviated comprehensive geriatric assessment (aCGA) at baseline and after 6 months of follow-up and indication for frailty according to aCGA among participants with protein-energy malnutrition (PEM) or its risk.

DOMAINS of frailty according aCGA	Cut-off value	Intervention group (*n* = 90)		*P*-value	Control group (*n* = 55)		*P*-value
		Baseline prevalence *n* (%)	6 mo prevalence n (%)		Baseline prevalence *n* (%)	6 mo prevalence *n* (%)	
Functional ability	≥1	74 (82.2)	75 (83.3)		51 (92.7)	53 (96.4)	
Bathing (ADL)		33 (36.7)	10 (11.1)		23 (41.8)	7 (12.7)	
Transferring (ADL)		13 (14.4)	2 (2.2)		2 (3.6)	1 (1.8)	
Continence (ADL)		40 (44.4)	44 (48.9)		23 (41.8)	20 (36.4)	
Shopping (IADL)		42 (46.7)	23 (25.6)		37 (67.3)	26 (47.3)	
Preparing food (IADL)		56 (62.2)	48 (53.3)		34 (61.8)	39 (70.9)	
Housekeeping (IADL)		25 (27.8)	33 (36.7)		17 (30.9)	21 (38.2)	
Laundry (IADL)		37 (41.1)	35 (38.9)		27 (49.1)	36 (65.5)	
Cognitive symptoms (MMSE)	≤6	54 (60.0)	61 (67.8)		30 (54.5)	39 (70.9)	
Attention and Calculation		13 (14.4)	26 (28.9)		11 (20.0)	10 (18.2)	
Reading		11 (12.2)	11 (12.2)		8 (14.5)	9 (16.4)	
Writing		15 (16.7)	27 (30.0)		16 (29.1)	15 (27.3)	
Copying		32 (35.6)	34 (37.8)		24 (43.6)	24 (43.6)	
Depressive symptoms (GDS-15)	≥2	53 (58.9)	7 (7.8)		25 (45.5)	13 (23.6)	
Emptiness		23 (25.6)	20 (22.2)		11 (20.0)	15 (27.3)	
Happiness		12 (13.3)	10 (11.1)		4 (7.3)	6 (10.9)	
Helpless		31 (34.4)	33 (36.7)		29 (52.7)	26 (47.3)	
Worthless		27 (30.0)	19 (21.1)		21 (38.2)	19 (34.5)	
Definition of frailty according aCGA		67 (74.4)	55 (61.1)	<0.001	41 (74.5)	44 (80.0)	0.291

*mo* month, *ADL* Activities of Daily Living (Barthel Index), *IADL* Instrumental Activities of Daily Living, *MMSE* Mini Mental State Examination, *GDS-15* Geriatric Depression Scale.

## Discussion

To our knowledge, our study is the first to examine the effects of individually tailored nutritional counseling on persons with PEM and frailty, including common problems in cognition, functional abilities, and unable to take care of themselves including their nutrition. We found that individual nutritional counseling, which focused on increasing protein and energy intake while considering individual dietary preferences, was associated with a decrease in frailty status among older home care clients with PEM or its risk. Our findings are consistent with previous research indicating that higher protein intake is inversely associated with frailty in older adults [31, 32]. Nutritional status is crucial to all frailty criteria [2], and a higher protein intake can slow down the decline of muscle mass, walking speed, and weight loss [33]. Among frail older persons, higher protein intake is associated with better muscle mass and physical performance, which can improve function ability [33, 34]. Moreover, our study found that energy intake is also associated with a decrease in frailty. Adequate dietary energy is essential for protein to effectively stimulate muscle protein synthesis and prevent muscle mass loss [35]. Therefore, our findings suggest that both protein and energy intake are critical for reducing frailty among older people, as supported by previous research [32, 36, 37].

It seems, that dietary intervention targeting sufficient protein and energy intake can have an impact on other symptoms, including a decrease in depressive symptoms according to the aCGA. This could be due to increased social interaction related to nutrition, which is supported by previous studies that have shown improved quality of life, self-rated health, cognitive function, and fewer depressive symptoms as a result of nutrition intervention [38, 39]. Furthermore, our findings indicate that ensuring adequate protein and energy intake is a viable strategy for preventing and reducing frailty in older people, even in vulnerable populations receiving home care. Since frailty and PEM are closely associated [13], treating PEM and improving nutritional status [16] can explain the reduction in frailty among participants in the intervention group.

Strengths of our study include its real-life intervention among home care clients, without any exclusion criteria. The study’s multidisciplinary approach and use of validated instruments also contributed to its value. Additionally, the individually tailored nutritional intervention based on participants’ preferences likely enhanced its effectiveness and acceptance. Furthermore, the nutrition intervention was administered by the same registered nutritionist, which enhanced reliability. We collected comprehensive data on nutrient intake using the 24-h recall method, administered by a registered nutritionist, and the information on cognitive decline was obtained from medical records, family caretakers, and homecare nurses. The study’s limitation was the short follow-up of only six months. However, home care clients’ health status is often unstable, and a longer follow-up could have resulted in a higher loss of participants.

## Conclusion

An individual-tailored nutritional intervention can decrease the prevalence of frailty status among vulnerable home care clients with PEM or its risk. In the nutritional treatment of frailty, adequate intake of protein and energy should be a cornerstone of treatment. By addressing individual nutritional needs, we can help vulnerable home care clients improve their quality of life and overall health.

## Supplementary information

Supplemental Table 1

## Data Availability

The datasets generated and/or analyzed during the current study are not publicly available due to limitations of ethical approval involving the patient data and anonymity but are available from the corresponding author on reasonable request.
