# Supplementary material for: Effect of individually tailored nutritional counseling on frailty status in older adults with protein-energy malnutrition or risk of it: an intervention study among home care clients
Source: Eur J Clin Nutr. 2024 Nov 23;79(4):306–10. doi: 10.1038/s41430-024-01547-0 (PMC11981931; doi:10.1038/s41430-024-01547-0)
Supplement: Supplementary file 1 — Supplemental Table 1 [file 41430_2024_1547_MOESM1_ESM.docx]

**Supplemental Table 1.** Definition of frailty according to abbreviated

comprehensive geriatric assessment (aCGA) among participants

with protein-energy malnutrition (PEM) or its risk.

| **DOMAINS of frailty**  **according aCGA** | **Cut-off value** |
| --- | --- |
|  |  |
| **Functional ability** | $\boldsymbol{\geq}$**1** |
| Bathing (ADL) |  |
| Transferring (ADL) |  |
| Continence (ADL) |  |
| Shopping (IADL) |  |
| Preparing food (IADL) |  |
| Housekeeping (IADL) |  |
| Laundry (IADL) |  |
|  |  |
| **Cognitive symptoms (MMSE)** | $\boldsymbol{\leq}$**6** |
| Attention and Calculation |  |
| Reading |  |
| Writing |  |
| Copying |  |
|  |  |
| **Depressive symptoms (GDS-15)** | $\boldsymbol{\geq}$**2** |
| Emptiness |  |
| Happiness |  |
| Helpless |  |
| Worthless |  |
|  |  |
| **Definition of frailty**  **according aCGA** | **Positive score on ≥2 domain** |

ADL=Activities of Daily Living (Barthel Index);

IADL=Instrumental Activities of Daily Living;

MMSE=Mini Mental State Examination;

GDS-15=Geriatric Depression Scale
